# High intellectual ability and the gut-brain-sex steroids axis: a perspective on cognitive and emotional diversity

**DOI:** 10.3389/fphys.2026.1791778

**Published:** 2026-04-13

**Authors:** Ítalo M. Urrutia, Nicolás Plaza, Felipe Moraga, Constanza Griffiths-Sanhueza, Diliana Pérez-Reytor, Eduardo Karahanian, Sebastián Ramírez-Araya, Ana Kinkead, María Paz Gómez, Katherine Garcia

**Affiliations:** 1 Instituto de Ciencias Biomédicas, Facultad de Ciencias de la Salud, Universidad Autónoma de Chile, Santiago, Chile; 2 Molecular Biology Laboratory, Masblue Research and Innovation Center, Iquique, Chile; 3 Escuela de Psicología, Universidad de Santiago de Chile, Santiago, Chile

**Keywords:** gut microbiota, gut-brain axis, high intellectual ability, neurodevelopment, neurodiversity, sex hormones, steroid metabolism

## Abstract

The gut-brain axis is a bidirectional communication network integrating neural, endocrine, immune, and metabolic signals that regulate neurodevelopment, cognition, and emotion. It contributes to neurotransmitter production, inflammatory regulation, and the microbial metabolism of sex steroids, processes that have been shown to modulate synaptic plasticity and emotional behavior in experimental and clinical contexts, although their specific relevance to high intellectual ability remains unknown. In this perspective, we propose that high intellectual ability could be explored as a heterogeneous construct, within which some individuals identified as having HIA may exhibit responses potentially associated with differential sensitivity to gut-brain-sex hormone interactions. We discuss that microbial modulation of steroid bioavailability and neuroactive metabolites may represent one hypothetical pathway through which variations in steroid bioavailability and neuroactive metabolites could intersect with cognitive performance and emotional intensity, traits frequently described in some individuals with HIA. Integrating evidence from neuroendocrinology, microbiome science, and cognitive neuroscience, we outline a conceptual framework linking microbial, hormonal, and neural processes. This model aims to stimulate empirical research examining how physiological variation across the gut-brain-sex hormone axis may underlie cognitive and emotional diversity in gifted subpopulations. Importantly, this framework is conceptual and extrapolates from converging evidence in microbiome science and neuroendocrinology, as direct empirical studies in high intellectual ability are currently lacking.

## 1 Introduction

The gut-brain axis is a bidirectional network integrating neural, endocrine, immune, and metabolic signals that regulate neurodevelopment, cognition, and emotion (Mayer et al., 2014; McEwen, 2017). Within this system, the gut microbiota modulates neuroactive metabolite production, inflammatory tone, and sex steroid availability through enzymatic pathways such as β-glucuronidases, reductases, and dehydrogenases (Dinan and Cryan, 2017; Plaza-Díaz et al., 2017; Gambaro et al., 2020; Loh et al., 2024). These processes influence neurotransmission, synaptic plasticity, and hypothalamic-pituitary-gonadal signaling in experimental and clinical contexts (Dinan and Cryan, 2017; Cryan et al., 2020), although their relevance to high intellectual ability (HIA) has not been empirically tested. In parallel, estrogens and androgens exert organizational and activational effects on learning, memory, executive function, and social cognition across development (Baron-Cohen et al., 2005; McCarthy et al., 2012; Luine, 2014; McEwen and Akil, 2020). Despite advances in microbiome science and neuroendocrinology, these pathways have rarely been considered in HIA research, which typically emphasizes cognitive potential shaped by creativity, motivation, and environmental factors (Renzulli, 2005; Gagné, 2005; Subotnik et al., 2011). Notably, recent studies link gut microbiome composition and function to neurodevelopment, functional brain connectivity, and emotional regulation in infancy and childhood (Petrut et al., 2025; Querdasi et al., 2025), supporting the biological relevance of the gut-brain axis and providing a physiological rationale for future research in HIA subpopulations.

In this Perspective, we use the term HIA to refer to individuals who consistently demonstrate advanced cognitive performance relative to age-matched peers (Subotnik et al., 2011; Conejeros-Solar et al., 2025), most commonly identified through psychometric assessment, but also associated with distinctive patterns of cognitive engagement and emotional functioning. Importantly, HIA extends beyond high intelligence quotient (IQ) scores or academic achievement (Subotnik et al., 2011). Additionally, the characterization of heightened emotional intensity as a defining feature of HIA remains debated, particularly given the limited empirical support for widely circulated characteristics lists in gifted education (Makel, 2025). Contemporary research increasingly suggests that emotional dysregulation or heightened reactivity observed in some gifted populations may often reflect contextual mismatch, educational under-stimulation, social experiences, or co-occurring neurodevelopmental conditions, including twice-exceptional profiles, rather than intrinsic neurobiological traits (Foley-Nicpon et al., 2011; Francis et al., 2016). Accordingly, the present framework does not assume that emotional intensity is a universal or defining characteristic of HIA. Instead, we propose that physiological sensitivity within the gut-brain-steroid axis may interact with environmental and developmental factors in specific subgroups, contributing to variability in cognitive engagement and emotional expression rather than constituting a core trait of giftedness itself.

Children with HIA often display rapid learning, advanced reasoning, curiosity, creativity, and asynchronous development (Peters et al., 2014; Worrell et al., 2019). Neuroimaging of individuals with higher general intelligence, overlapping with high-ability populations, shows greater neural efficiency, stronger structural connectivity, and enhanced network integration (Neubauer and Fink, 2009; Hilger et al., 2017). However, the biological processes underlying the distinctive cognitive and emotional profiles of HIA in some individuals remain largely unexplored. Identification is hindered by narrow criteria, gender bias, and behavioral misinterpretation, disproportionately affecting disadvantaged or culturally diverse students (Bianco et al., 2011; Subotnik et al., 2011). Giftedness is now viewed as a heterogeneous, multidimensional expression shaped by contextual and intrapersonal factors, yet current models rarely consider physiological systems, particularly microbiota-steroid interactions, that may explain observed variability and intensity.

Studies by Celec, Durdiakova, Ostatníková, and colleagues report genetic variants affecting androgen and estrogen signaling in gifted children: ESR2 polymorphisms influencing ERβ in boys, variants affecting testosterone metabolism (Celec et al., 2015), associations between AR CAG repeat length and visuospatial performance, links between testosterone metabolism and non-verbal IQ in girls (Durdiakova et al., 2011), and salivary testosterone correlations with cognitive performance (Ostatnikova et al., 2007). These findings indicate preliminary and methodologically limited evidence for altered neuroendocrine responsivity, derived from small and heterogeneous samples.

Although some HIA individuals may show elevated cognitive potential with greater emotional sensitivity, no integrative framework examines interactions among microbial steroid metabolism, neuroendocrine signaling, and neural plasticity. This gap limits understanding of the biological factors underlying cognitive and emotional heterogeneity. To our knowledge, no model integrates microbiome function, sex steroid sensitivity, and neurodevelopment to explain these variations.

## 2 Core argument: the gut-brain-steroids axis in high intellectual ability

HIA is typically understood from psychological and educational perspectives, emphasizing cognitive performance, socioemotional traits, and learning trajectories. However, these approaches offer limited insight into the biological processes that may contribute to the different profiles observed in this population. Here, we advance a conceptual model proposing that certain subgroups of individuals identified as having HIA, particularly those demonstrating both elevated cognitive performance and heightened emotional sensitivity, could be explored in relation to variability within the gut-brain-steroid (GBS) axis. This model is based on the extrapolation of three independent but converging lines of evidence: (1) microbial regulation of sex steroid metabolism, (2) established roles of estrogens and androgens in neurodevelopment and cognition, and (3) preliminary findings suggesting altered steroid receptor sensitivity in some intellectually gifted children. Importantly, this integrative framework remains hypothetical and has not been directly tested in HIA cohorts. First, the gut microbiota regulates the availability and transformation of sex hormones through enzymatic pathways such as β-glucuronidase, reductases, and dehydrogenases (Plaza-Díaz et al., 2017), processes that have been shown in experimental and clinical contexts to modulate synaptic plasticity, neurotransmission, and aspects of neurodevelopment. Second, sex steroids exert profound organizational and activational effects on learning, memory, and executive functions (McCarthy et al., 2012; Luine, 2014). Third, a few studies in intellectually gifted children suggest altered sensitivity to both androgens and estrogens, including differences in ESR2 and androgen-related polymorphisms associated with cognitive performance (Durdiakova et al., 2011; Celec et al., 2015). If neural responsiveness to steroid signaling differs in some individuals with HIA, as suggested by receptor polymorphism studies (Durdiakova et al., 2011; Celec et al., 2015), then variability in microbial steroid metabolism could represent a modulatory factor that interacts with cognitive and emotional processes. Whether such interactions are quantitatively or qualitatively distinct in HIA remains unknown and requires empirical investigation. In this view, variability in the GBS axis may modulate the phenotypic expression of cognitive and emotional traits commonly reported in some HIA subpopulations, positioning giftedness as a form of neurobiological diversity influenced by microbial, endocrine, and neural interactions.

We emphasize that the microbiota-steroid-cognition pathway described here does not imply a linear or deterministic mechanism. Rather, it represents a systems-level model in which microbial metabolism may interact with endocrine and neural processes within broader genetic, developmental, and environmental contexts. Direct evidence testing this pathway in individuals with HIA is currently lacking.

## 3 GBS axis and sex steroids: modulating systems of the development of cognitive and emotional function

Human cognitive and emotional variability emerges from interaction among genetic, environmental and educational factors, as well as integrated physiological systems shaping brain development. Two of the most influential systems are the gut-brain axis and sex-steroid signaling, both regulating neural plasticity, neurotransmission, and stress responsivity (Mayer et al., 2014; McEwen, 2017). The gut microbiota modulates neurogenesis, neurotransmitter production and systemic inflammatory tone (Dinan and Cryan, 2017; Gambaro et al., 2020), influencing cognitive and affective processing from early development through adolescence, periods may be associated with high plasticity (Dinan and Cryan, 2017; Cryan et al., 2020).

Crucially, the microbiota also influences steroid hormone availability and transformation, positioning microbes not only as neuromodulators but also as modulators of endocrine signaling. Enzymatic activities such as microbial β-glucuronidases contribute to the deconjugation of estrogen metabolites in the intestinal lumen, facilitating enterohepatic recirculation and influencing systemic estrogen availability (Hu et al., 2023). Additional microbial enzymes, including reductases and dehydrogenases, have been implicated in steroid biotransformation pathways (Plaza-Díaz et al., 2017). Through these mechanisms, the gut microbiota may affect circulating and locally active steroid levels. Beyond steroids, gut bacteria synthesize neuroactive compounds, including serotonin, dopamine and GABA (Bercik et al., 2011; Yano et al., 2015), and produce short-chain fatty acids (SCFAs) that influence neuroprotection, blood-brain barrier integrity, and the expression of synaptic genes (Borre et al., 2014).

Taken together, these mechanisms support the conceptualization of a GBS axis integrating microbial metabolism with endocrine and neural development. This axis provides a conceptual framework for exploring interindividual differences in cognition and emotional regulation, although its specific contribution to variations in intellectual ability, if any, has not yet been determined. It also raises the possibility that populations with distinctive cognitive, such as individuals with HIA, could exhibit differential sensitivity to these physiological pathways, a hypothesis that remains to be empirically evaluated.

On the other hand, sex steroids exert organizational effects during neurodevelopment and activational effects across the lifespan. Estradiol enhances synaptic plasticity, dendritic remodeling and executive function (McCarthy et al., 2012; Luine, 2014), whereas testosterone contributes to neural lateralization, visuospatial processing and decision-making (Baron-Cohen et al., 2005; McEwen and Akil, 2020). These hormones act in multiple brain regions via receptor-specific mechanisms that interact with environmental, microbial and neural inputs.

Puberty represents a developmental window characterized by marked increases in estradiol and testosterone, which contribute to the reorganization of limbic-prefrontal circuitry involved in emotional regulation and executive control (Gunnar and Quevedo, 2007; Spear, 2009). Some adolescents identified with HIA have been described as exhibiting emotional intensity, although as we mentioned, such traits are neither universal nor specific to this population. Within the present conceptual framework, this developmental period provides a context in which differences in receptor sensitivity or endocrine responsiveness could, in theory, modulate typical maturational trajectories. This proposition remains hypothetical and generates a testable prediction: that subgroup-specific variability in steroid sensitivity may interact with pubertal endocrine changes to influence cognitive and emotional expression in certain individuals with HIA.

Recent experimental evidence indicates that alterations in female sex hormone status induce measurable shifts in gut microbial composition, which in turn exacerbate metabolic dysfunction, supporting bidirectional microbiota-steroid interactions (Cross et al., 2023).

Thus, sex steroids represent a key biological dimension through which the GBS axis may shape cognitive profiles. If microbial metabolism modulates steroid availability, and if individual brains differ in their responsiveness to these hormones, then the intersection of these systems could help explain variability in cognitive capacity and emotional depth, including the distinctive traits observed in HIA.

## 4 Neurobiological variability within HIA: endocrine sensitivity and microbiota-dependent modulation

HIA has traditionally been described through cognitive performance and psychological traits. Although a limited number of studies have examined psychophysiological and endocrine correlates in specific samples of intellectually gifted individuals (Correia et al., 2005), systematic physiological characterization of this population remains scarce. From a physiological standpoint, interindividual differences observed in these preliminary studies raise the possibility that variability in neural responsiveness to sex steroids and microbiota-derived metabolites during critical windows of brain development may contribute to cognitive and emotional diversity.

Sex steroids exert powerful organizational and activational effects on the developing brain through receptor-mediated mechanisms. Estradiol, acting predominantly via estrogen receptor β (ERβ), regulates synaptic plasticity, dendritic spine density, and executive network maturation, particularly within prefrontal and hippocampal circuits (McCarthy et al., 2012; Luine, 2014). Testosterone and its metabolites modulate neural lateralization, visuospatial processing, and stress responsivity through androgen receptor (AR)-dependent pathways (Baron-Cohen et al., 2005; McEwen and Akil, 2020). Recent human evidence further supports potential associations between gut microbiome composition and circulating testosterone levels. A systematic review of studies in men highlights correlations between specific microbial taxa and androgen concentrations, while also emphasizing substantial methodological heterogeneity and the need for longitudinal and mechanistic studies to clarify causality (Pakpahan et al., 2024). Importantly, genetic studies in intellectually gifted children report polymorphisms in ESR2, as well as variation in AR CAG repeat length, that correlate with cognitive performance, suggesting altered receptor-level sensitivity rather than differences in absolute hormone concentrations (Durdiakova et al., 2011; Celec et al., 2015). These findings derive from specific psychometrically identified child samples and should not be generalized to the broader heterogeneous construct of HIA.

Beyond endocrine signaling alone, the gut microbiota plays a modulatory role in steroid bioavailability through the estrobolome, a functional ensemble of microbial genes encoding β-glucuronidases and related enzymes that regulate estrogen deconjugation and reabsorption within the intestine (Hu et al., 2023). By influencing enterohepatic recirculation of estrogens, variability in estrobolome activity may contribute to differences in systemic estrogen exposure. In theory, such variability could have differential neural consequences in individuals exhibiting altered receptor sensitivity, although this possibility has not been empirically examined in any HIA populations.

In parallel, microbial production of SCFAs including acetate, propionate, and butyrate influences blood-brain barrier integrity, microglial maturation, and neurotrophic factor expression such as BDNF (Borre et al., 2014; Cryan et al., 2019). These pathways converge with steroid signaling at synaptic plasticity and stress regulation, providing a substrate through which microbiota composition could modulate cognitive and emotional processing in individuals exhibiting heightened endocrine sensitivity.

These interactions are likely to be developmentally constrained, with strongest effects during periods of heightened neural plasticity such as early childhood and puberty. Pubertal surges in estradiol and testosterone reorganize limbic-prefrontal circuitry involved in emotional regulation and executive control (Gunnar and Quevedo, 2007; Spear, 2009). If some HIA subgroups exhibit increased receptor or signaling sensitivity, microbial modulation of steroid availability during these windows could contribute to enhanced cognitive engagement and variability in emotional expression.

Under this framework, neurobiological variability within HIA can be explored as potentially involving dynamic interactions among microbiota-dependent metabolism, sex steroid signaling, and neural plasticity. This interpretation remains speculative and is intended to generate testable hypotheses rather than to describe an established biological mechanism. This perspective aims to offer a physiologically informed framework for investigating cognitive and emotional diversity within HIA subpopulations.

Importantly, HIA represents a heterogeneous construct encompassing individuals identified through diverse criteria, including psychometric performance, domain-specific talent, creativity, and contextual achievement (Subotnik et al., 2011; Worrell et al., 2019). The endocrine and genetic findings discussed here are based on specific child samples identified through psychometric criteria and should not be generalized to all high-ability populations. The proposed GBS framework may therefore be most applicable to subgroups characterized by the co-occurrence of elevated cognitive performance and heightened emotional intensity, rather than to HIA as a uniform construct.

## 5 Perspectives and future directions

This perspective proposes that some individuals identified as having HIA may exhibit heightened sensitivity within the GBS axis, providing a conceptual framework integrating microbial, endocrine, and neural processes. While the individual components of this framework are supported by substantial empirical evidence, including microbial metabolism of sex steroids, the neurobiological actions of estrogens and androgens, and the influence of microbial metabolites on neuronal plasticity (Plaza-Díaz et al., 2017; Cryan et al., 2019; Leao et al., 2025), their relevance to HIA remains largely unexplored. Future research should focus on mechanistic-developmental pathways, environmental moderators, and ethical considerations.

## 6 Biological and developmental pathways linking microbiota, endocrine sensitivity, and neural plasticity

**Figure 1:**
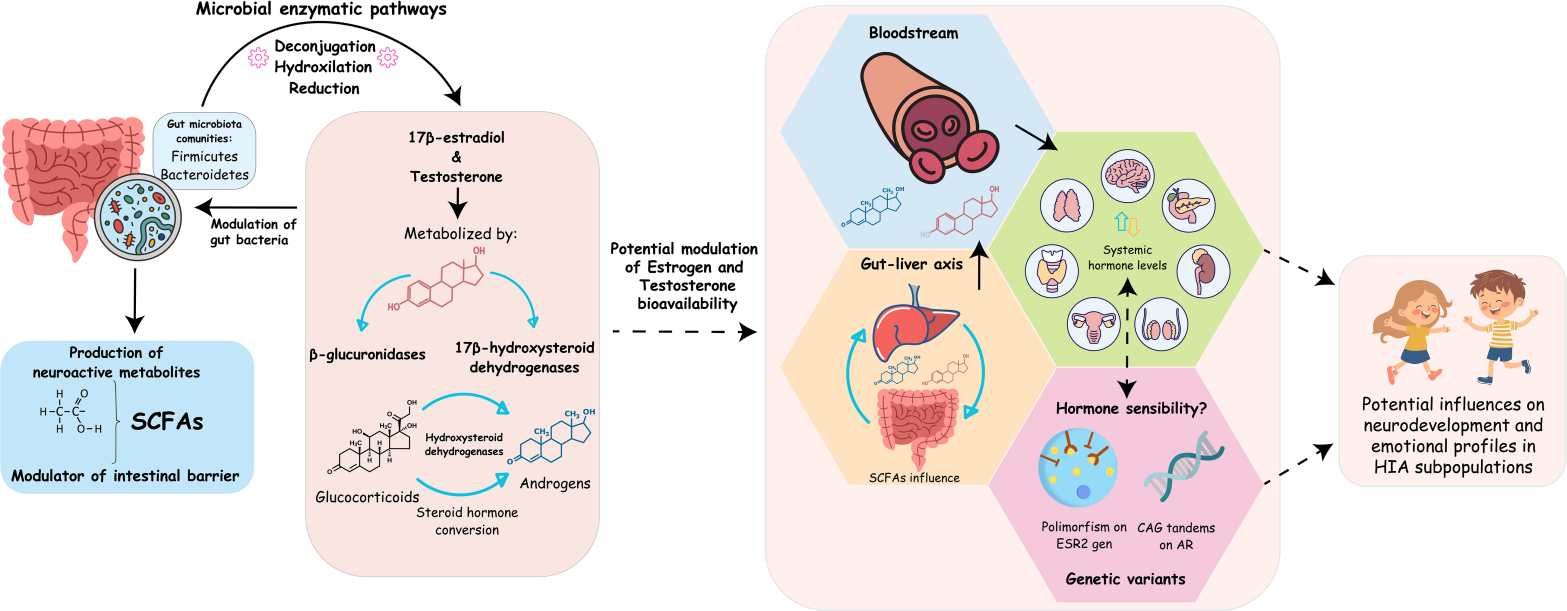
Conceptual model of the gut-brain-sex steroid (GBS) axis as a modulatory framework for neurodevelopmental variability. The figure illustrates how gut microbiota-dependent metabolic activities may interact with endocrine and neural systems to influence cognitive and emotional traits. Microbial enzymatic pathways (e.g., deconjugation, hydroxylation, reduction) can modulate the bioavailability of estrogens and androgens via the gut-liver axis and systemic circulation. In parallel, microbiota-derived metabolites such as short-chain fatty acids (SCFAs) act as modulatory signals influencing intestinal barrier function and metabolic signaling. Systemic hormone levels interact with interindividual differences in hormone sensitivity, shaped in part by genetic variants (e.g., ESR2 polymorphisms, androgen receptor CAG repeats). These interactions are proposed to be particularly relevant during developmental windows of heightened neural plasticity, potentially contributing to variability in neurodevelopmental and emotional profiles among HIA subpopulations. Dashed arrows indicate hypothesized or indirect modulatory interactions rather than established causal pathways. Elements shown are illustrative and not intended as specific biomarkers.

A key direction for future research is to elucidate how microbial sex steroid metabolism intersects with neuroendocrine dynamics across childhood and puberty. This requires characterizing gut microbial composition, estrobolome activity, androgen precursor transformation and circulating steroid levels in individuals with HIA (**Figure 1**). Integrated metagenomics, metabolomics, and endocrine profiling could determine whether microbial regulation of estrogen and androgen availability contributes to the cognitive, emotional, and physiological traits described in HIA. Such work would enable direct testing of whether microbial metabolites and sex steroids jointly shape neurocognitive variability.

These interactions should be considered within developmental windows of heightened neural plasticity. Childhood and puberty are critical periods during which microbiota-endocrine interactions may exert amplified effects on brain maturation. Pubertal surges in estradiol and testosterone reorganize limbic and prefrontal circuits involved in emotional regulation, motivation, and executive function (Gunnar and Quevedo, 2007; Spear, 2009). If some individuals identified as HIA exhibit increased endocrine sensitivity, as suggested by preliminary genetic and hormonal findings, microbial modulation of steroid signaling may be particularly consequential during these neurodevelopmental phases.

Longitudinal studies integrating functional neuroimaging, microbiome sequencing and repeated endocrine assessments could clarify whether highly capable brains exhibit distinctive trajectories of hormonal responsiveness or circuit-level reorganization in response to microbial and endocrine cues. Developmentally informed studies must incorporate sex-specific analyses, given established differences in estrogenic and androgenic responsivity across male and female developmental trajectories (Sullivan and Ciernia, 2022; VanRyzin et al., 2020). Recognizing these biological nuances is essential for identifying when and how the GBS axis may strongly influence cognitive and emotional expression.

## 7 Environmental moderators and opportunities for non-invasive support

Beyond biological mechanisms, environmental factors that shape the gut microbiota represent an important area for future research. Diet, sleep, stress, antibiotic exposure and physical activity influence microbial composition and metabolite production (Cryan et al., 2019). If some individuals with HIA are more sensitive to fluctuations in the GBS axis, these factors may strongly affect cognitive performance, emotional sensitivity, and physiological regulation.

**Figure 2:**
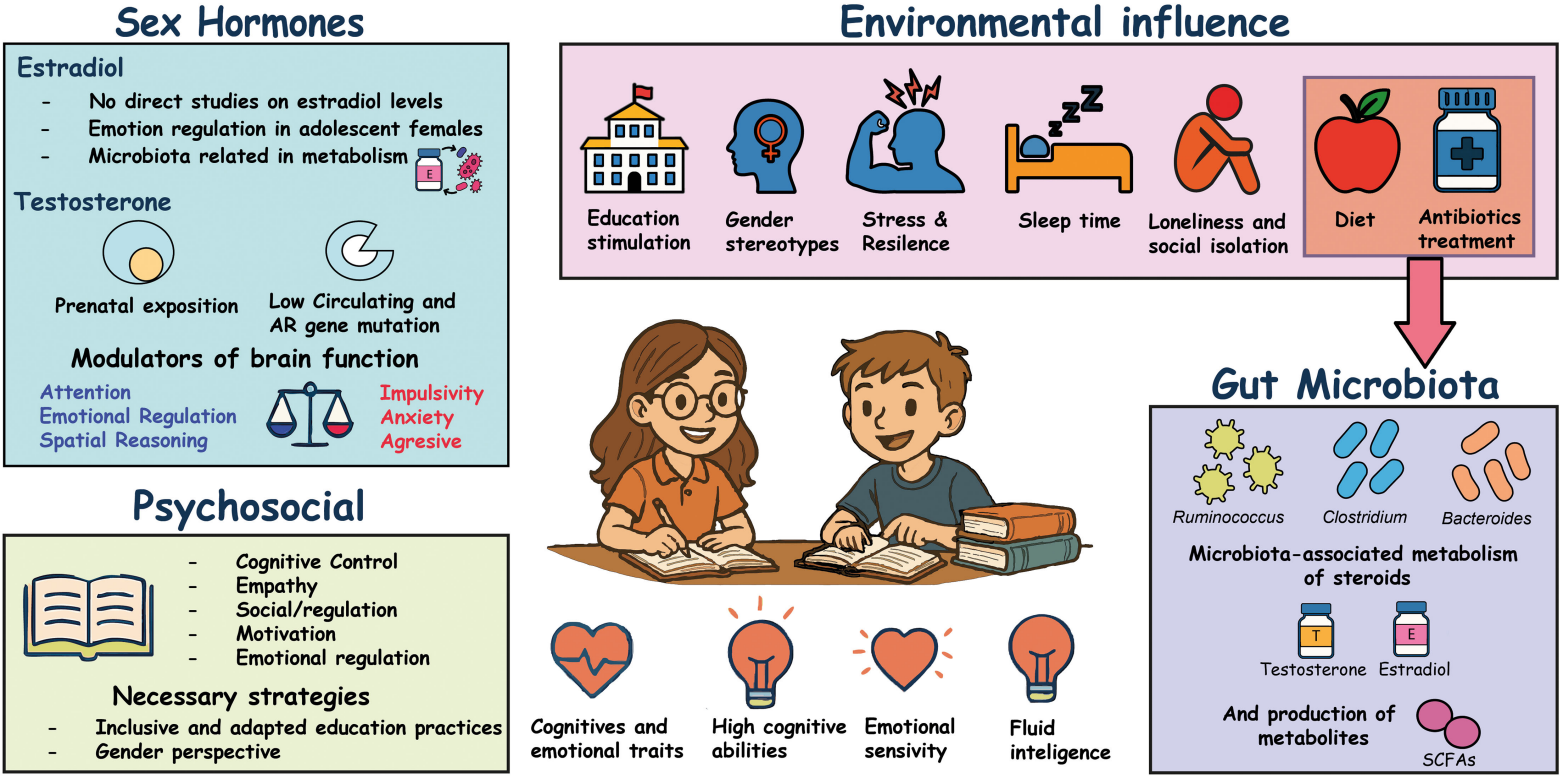
Environmental, psychosocial, and biological factors interacting with the GBS axis in high intellectual ability. Integrated conceptual perspective depicting how environmental and psychosocial factors may interact with the gut-brain-sex steroid (GBS) axis to shape cognitive and emotional trait expression in individuals with high intellectual ability (HIA). Environmental influences such as diet, antibiotic exposure, sleep patterns, stress, and social context can modulate gut microbiota composition and metabolic activity, indirectly influencing host-derived steroid hormones (T and E) through microbiota-associated enzymatic and signaling mechanisms, as well as the production of microbial metabolites such as SCFAs. Psychosocial dimensions, including emotional regulation, motivation, empathy, and cognitive control, interact bidirectionally with biological systems across development. Together, these factors may contribute to variability in cognitive performance, emotional sensitivity, and adaptive functioning. This perspective emphasizes non-deterministic, modulatory interactions and does not imply direct hormonal synthesis by microbiota, causal relationships, cognitive enhancement, or biological optimization.

This suggests that non-pharmacological, microbiota-supportive strategies such as dietary fiber intake, stress reduction, sleep optimization, or prebiotic approaches, could promote microbial stability and indirectly support wellbeing (**Figure 2**). However, these approaches require rigorous evaluation and ethical caution and should be framed as supportive rather than cognitive enhancement.

## 8 Ethical and conceptual considerations

Exploring the interplay between microbiota, hormones, and intellectual functioning raises important ethical issues. A biological framework for HIA should not be viewed as deterministic or used to justify cognitive enhancement or hormonal manipulation. Rather, this perspective aims to complement existing psychological and educational models by incorporating physiological and developmental insights. Recognizing that some HIA subgroups may show increased biological sensitivity could help parents, educators, and clinicians provide more empathetic and evidence-informed support, while avoiding reductionism, overgeneralization, and societal misinterpretation.

## 9 Conclusion

We propose that specific subgroups within the heterogeneous construct of HIA warrant investigation under a systems-based physiological framework integrating microbiota, endocrine, and neural dimensions. At present, this model is inferential and requires direct empirical validation in well-characterized HIA populations. Exploring this model expands our understanding of human cognitive diversity and opens avenues for integrating biology, psychology, and education in support of the holistic development of individuals with HIA. This perspective represents a first step toward an interdisciplinary framework that acknowledges the complexity and uniqueness of psychobiological profiles associated with high ability, inviting future research to clarify the mechanisms connecting gut biology, endocrine regulation, and intellectual potential.

## Data Availability

The original contributions presented in the study are included in the article/supplementary material. Further inquiries can be directed to the corresponding author/s.
